# Gag Reflex-Mediated Restoration of Sinus Rhythm during TEE Probe Insertion for Atrial Fibrillation: A Word of Caution

**DOI:** 10.1155/2020/6398196

**Published:** 2020-03-02

**Authors:** Zeid Nesheiwat, Marcel Ghanim, Joseph Eid, Neha Patel, Cameron Burmeister, Ehab Eltahawy

**Affiliations:** ^1^Department of Internal Medicine, The University of Toledo Medical Center, Toledo, Ohio, USA; ^2^Department of Cardiology, The University of Toledo Medical Center, Toledo, Ohio, USA

## Abstract

It is recommended to attempt vagal maneuvers as initial therapy in various types of supraventricular tachycardia. While various forms of vagal techniques have been described, a gag reflex-mediated vagal technique, to the best of our knowledge, has not been. We present a case of gag reflex-mediated restoration of sinus rhythm in a patient with atrial fibrillation and rapid ventricular response upon transesophageal probe insertion. This case is unique due to the mechanism of vagally mediated cardioversion. It emphasizes that operators must be cautious regarding the risk of embolization of a potential thrombus from vagal-mediated cardioversion with unknown thrombus burden.

## 1. Introduction

Supraventricular tachycardias (SVTs) are narrow complex tachyarrhythmias with QRS complex of less than 100 milliseconds [1]. It has been shown that parasympathetic input to the heart results in slowing of the heart rate through various pathways. Parasympathetic stimulation causes the release of acetylcholine, which then slows the atrioventricular node conduction velocity while increasing the refractory period and decreasing the rate of impulse through the sinus node [2]. Vagal maneuvers are used as an initial attempt to decrease heart rate in SVTs.

Atrial fibrillation is an SVT associated with increased risk for atrial thrombus formation and embolization. Transesophageal echocardiogram (TEE) is frequently performed to rule out the presence of atrial thrombi before elective cardioversion of atrial fibrillation to reduce the risk of stroke upon restoration of normal sinus rhythm. The success and risk of less invasive maneuvers, such as vagal stimulation, has not been thoroughly explored.

## 2. Case Presentation

**Figure 1:**
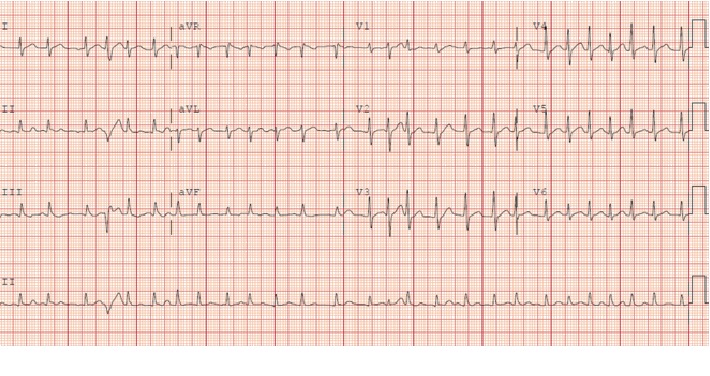
Precardioversion electrocardiogram showing atrial fibrillation with rapid ventricular response.

We present a 44-year-old male with no significant past medical history who arrived at the emergency department with sudden onset retrosternal chest pain of a 4-hour duration. The chest pain was described as heavy in nature and nonradiating, with associated dyspnea, diaphoresis, and dizziness. At initial evaluation, the patient was hemodynamically stable. Initial electrocardiogram revealed sinus tachycardia with no ischemic changes. Troponins were negative. However, during his stay, the patient suddenly developed palpitations and was found to be in atrial fibrillation with rapid ventricular response with a heart rate ranging from 130 to 150 (Figure 1).

The patient was initially placed on a diltiazem infusion to control the heart rate. The patient then underwent an echocardiogram which was unremarkable. A subsequent cardiac catheterization revealed severe, thrombotic stenosis of the mid left anterior descending coronary artery, which was successfully treated by balloon angioplasty and SYNERGY drug-eluting stent placement.

**Figure 2:**
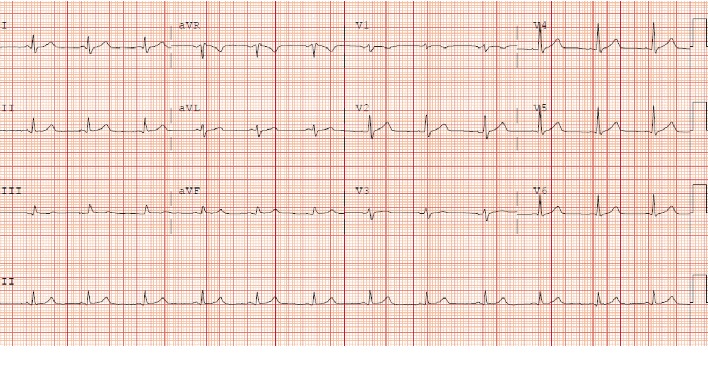
Post gag reflex-mediated cardioversion electrocardiogram showing normal sinus rhythm.

The following morning, the patient was scheduled for a transesophageal echocardiogram in anticipation of elective cardioversion as he remained in atrial fibrillation. Prior to the procedure, he received midazolam injection, topical butamben-tetracaine-benzocaine (CETACAINE) spray to the oropharynx, and propofol intravenously. Upon introduction of the TEE probe, the patient had several episodes of severe gagging following aggressive premedication. His rhythm then spontaneously reverted to sinus and no electrical cardioversion was necessary (Figure 2). TEE was completed and confirmed as having no valvular abnormalities or thrombus in the left atrial appendage. The patient maintained a normal sinus rhythm and was asymptomatic for the rest of his stay. The patient was discharged home in stable condition with follow-up in the cardiology clinic.

## 3. Discussion

Vagal maneuvers are relatively safe and easily performed techniques. Although mainly considered therapeutic for atrioventricular reentrant tachycardia (AVRT) and atrioventricular nodal reentry tachycardia (AVNRT), vagal techniques are recommended as an initial therapeutic option in patients with any form of SVT, although some studies have not shown sufficient evidence to support or refute their effectiveness [2–4]. In addition to slowing conduction through the AV node, vagal maneuvers have also been known to contribute to atrial fibrillation resolution by reducing P-wave dispersion. Several studies have concluded that large P-wave dispersion is associated with recurrences of atrial fibrillation after cardioversion [5].

In the setting of newly diagnosed atrial fibrillation and unknown thrombus burden, it is important to be aware of the potential risk of embolization from vagal-mediated cardioversion. Introduction of the TEE probe, despite aggressive premedication, can lead to a severe gag reflex and vagal stimulation. Although there are current guidelines in place for sedation and anesthesia during TEE, these guidelines do not address dosages [6]. Therefore, in order to minimize this risk, operators should be conscious, providing adequate pharyngeal anesthesia, sedation, and adequate time for the sedation to help decrease the gag reflex stimulation.

## 4. Conclusion

Vagal maneuvers are recommended as an initial therapeutic option for SVTs before medical or electrical intervention is performed. Believed to be relatively safe, vagal maneuvers are widely used. However, vagally induced cardioversion of SVT and in particular atrial fibrillation carries a risk of embolization; this is especially relevant in situations of inadequate anticoagulation and unknown thrombus burden.

Operators must be cautious regarding the risk of embolization of a potential thrombus from vagal-mediated cardioversion with unknown thrombus burden in patients with newly diagnosed atrial fibrillation.
